# Self-compassion interventions for family caregivers of people with dementia: a scoping review

**DOI:** 10.1590/0034-7167-2025-0252

**Published:** 2026-08-21

**Authors:** Marina Noto Faria, Joyce Salgado Santos, Fernanda Guilhermino Magalhães, Ana Karla Silva Soares, Ana Lucia de Moraes Horta

**Affiliations:** I Universidade Federal de São Paulo. São Paulo, São Paulo, Brazil; II Universidade Federal do Mato Grosso do Sul. Campo Grande, Mato Grosso do Sul, Brazil

**Keywords:** Dementia, Caregivers, Empathy, Psychosocial Intervention, Scoping Review., Demencia, Cuidador, Compasión, Intervenciones Psicosociales, Revisión de Alcance.

## Abstract

**Objectives:**

to identify interventions with the potential to foster self-compassion among family caregivers of people with dementia.

**Methods:**

we conducted a scoping review across eight databases using the search terms *dementia, self-compassion, empathy, family, caregivers, and mental health*. We included English-language articles addressing mindfulnessand/or compassion-based interventions. Three reviewers performed study selection with support from the Rayyan software.

**Results:**

of the 280 records identified, six studies were included. Interventions involved yoga, mindfulness, Acceptance and Commitment Therapy, self-compassion-based therapy, and mentalization. Three studies reported increased self-compassion.

**Conclusions:**

the literature suggests that some interventions may support the development of self-compassion among caregivers of people with dementia.

## RESUMEN

**Objetivos:**

identificar intervenciones con potencial para promover la autocompasión en familiares cuidadores de personas con demencia.

**Métodos:**

se realizó una revisión de alcance en ocho bases de datos, utilizando los términos de búsqueda *dementia, self*-*compassion, empathy, family, caregivers* y *mental health*. Se incluyeron estudios en inglés sobre intervenciones basadas en atención plena y/o compasión. Tres revisores realizaron la selección con apoyo del software Rayyan.

**Resultados:**

de 280 registros identificados, se incluyeron 6 estudios. Las intervenciones abarcaron yoga, mindfulness, Terapia de Aceptación y Compromiso, terapia basada en autocompasión y mentalización. Tres estudios informaron un aumento de la autocompasión.

**Conclusiones:**

la literatura sugiere que algunas intervenciones podrían promover la autocompasión en familiares cuidadores de personas con demencia.

## INTRODUCTION

The Diagnostic and Statistical Manual of Mental Disorders (DSM-5) classifies dementia as a neurodegenerative disease characterized by progressive cognitive decline, resulting in a significant reduction in intellectual and cognitive ability^(1)^. Cognitive decline can impair the skills needed to perform activities of daily living. As the disease worsens, people with dementia become increasingly dependent on others for care^(2)^.

Family members often serve as the primary caregivers for people with dementia^(2)^. Accordingly, increasing caregiving demands can have substantial consequences for these caregivers’ health. This population may experience emotional strain, fatigue, sleep disturbances, anxiety, depression, elevated stress levels, back pain, caregiver burden, and stigma^(2-6)^.

In response, multiple studies have addressed these family caregivers’ needs. A key need identified was the opportunity to share their experiences, concerns, and sources of satisfaction related to the caregiving process, as well as the need to develop coping strategies to improve physical and mental health^(2,7-9)^.

Self-compassion is a skill associated with well-being and with reductions in negative self referential thoughts, anxiety, depression, stress, rumination, avoidance, thought suppression, and shame, as well as a reduced psychological impact of internalized stigma^(10-17)^. Conceptually, it is sensitivity to one’s own suffering, accompanied by a commitment to try to alleviate it^(18,19)^. It comprises three pillars: self-kindness, mindfulness, and common humanity^(18)^. *Self-kindness* refers to the ability to be caring toward oneself amid suffering; *mindfulness* involves being aware of challenging thoughts and feelings rather than over-identifying with them^(20)^; and *common humanity* is the recognition of interconnectedness among human beings^(19)^.

As knowledge about self-compassion and its mental health benefits has expanded, national and international studies have applied mindfulness and other self-compassion-based practices among family members of people with dementia; however, few reviews address the impact of such interventions on this population’s mental health^(14,15)^.

## OBJECTIVES

To identify interventions with the potential to foster self-compassion among family caregivers of people with dementia.

## METHODS

This scoping review was conducted in accordance with JBI methodology for scoping reviews and is reported following the PRISMA-ScR checklist^(21,22)^. The review question was: “What types of interventions are available that have the potential to foster self-compassion among family caregivers of people with dementia, and what are their impacts on caregivers’ mental health?”. The protocol was registered in the Open Science Framework (OSF) and is available at OSF.IO/9P8RV (doi: 10.17605).

This scoping review followed these steps: defining the research question; defining keywords and the search strategy; selecting studies; and assessing full-text articles for eligibility. Eight databases were searched: PubMed (National Center for Biotechnology Information), the Virtual Health Library (BVS), EMBASE, ProQuest, PsycINFO, Latin American and Caribbean Health Sciences Literature (LILACS), the Education Resources Information Center (ERIC), and the Cumulative Index to Nursing and Allied Health Literature (CINAHL). Database-specific filters were applied, such as full-text availability, original research articles, and human studies. Studies published in English were included. Records without full-text access and gray literature were not included. No date restrictions were applied. The search was conducted between December 2023 and September 2024.

The search included controlled vocabulary terms for each database (DeCS and MeSH): dementia, self-compassion, empathy, compassion, family, caregivers, and mental health. The search strategy used to identify records was: (“Dementia”) AND (“caregivers”) AND (“Family” OR “partner” OR “family members” OR “members” OR “relatives”) AND (“self-compassion” OR “empathy” OR “compassion” OR “mindfulness” OR “mindfulness based stress reduction” OR “mindfulness based stress reduction” OR “mindfulness-based cognitive therapy” OR “acceptance and commitment therapy” OR “meditation” OR “loving kindness meditation*” OR “compassion meditation” OR “mindful self-compassion” OR “compassion focused therapy” OR “compassion cultivation training” OR “cultivating emotional balance”).

### Inclusion criteria

(1) original, full-text articles published in English in peer-reviewed journals; (2) studies on interventions involving compassion or self-compassion among family members of people with dementia; (3) samples composed exclusively of family caregivers of people with dementia, or family caregivers and the person/people with dementia; (4) studies assessing psychological outcomes (levels of compassion, self-compassion, anxiety, depression, caregiver burden, quality of life, well-being, among others) among family members of people with dementia; and (5) randomized and nonrandomized controlled designs, experimental and quasi-experimental studies, as well as qualitative studies. Eligible studies had to involve family members of people with dementia and include a mindfulnessand/or compassion-based intervention.

### Exclusion criteria

(1) studies published as letters, Master’s theses, theoretical articles, extended abstracts, and/or doctoral dissertations; and (2) studies that did not include preand post-intervention assessment (except for qualitative studies).

### Data collection, management, and analysis

Two independent reviewers conducted study selection and screening. References were managed in Rayyan^(23)^, and duplicate records were removed. Titles and abstracts were then screened; records that did not meet the eligibility criteria were excluded, with reasons for exclusion recorded. Next, the reviewers independently assessed full texts. Biweekly meetings were held throughout the screening process to ensure consistent decision-making. Disagreements were resolved by consensus and, when necessary, a third reviewer was consulted.

Interrater agreement was assessed using Cohen’s kappa, calculated in Jamovi^(24)^. Kappa was interpreted using the following categories: slight (up to 0.20); fair (0.21-0.40); moderate (0.41-0.60); substantial (0.61-0.80); and almost perfect (> 0.81)^(25,26)^.

To assess risk of bias and methodological quality, we used the JBI Critical Appraisal Tools. For study designs not covered by JBI appraisal tools, we used EQUATOR Network checklists. The JBI Critical Appraisal Tools include a set of questions about each article’s methods, with response options of “Yes”, “No”, “Unclear”, or “Not applicable”^(21)^. Based on these responses, methodological quality can be appraised: a higher number of “Yes” responses indicates better methodological quality, whereas more “No” or “Unclear” responses indicate lower adherence to the required methodological standards^(27)^. Among the EQUATOR Network checklists, STROBE (Strengthening the reporting of observational studies in epidemiology) was used to assess reporting completeness for one article^(28)^. STROBE is a 22-item reporting guideline intended to standardize reporting and improve transparency. The more items met, the more complete the reporting. However, the article assessed using STROBE was excluded because it did not meet the established methodological requirements^(22)^.

## RESULTS

**Figure 1:**
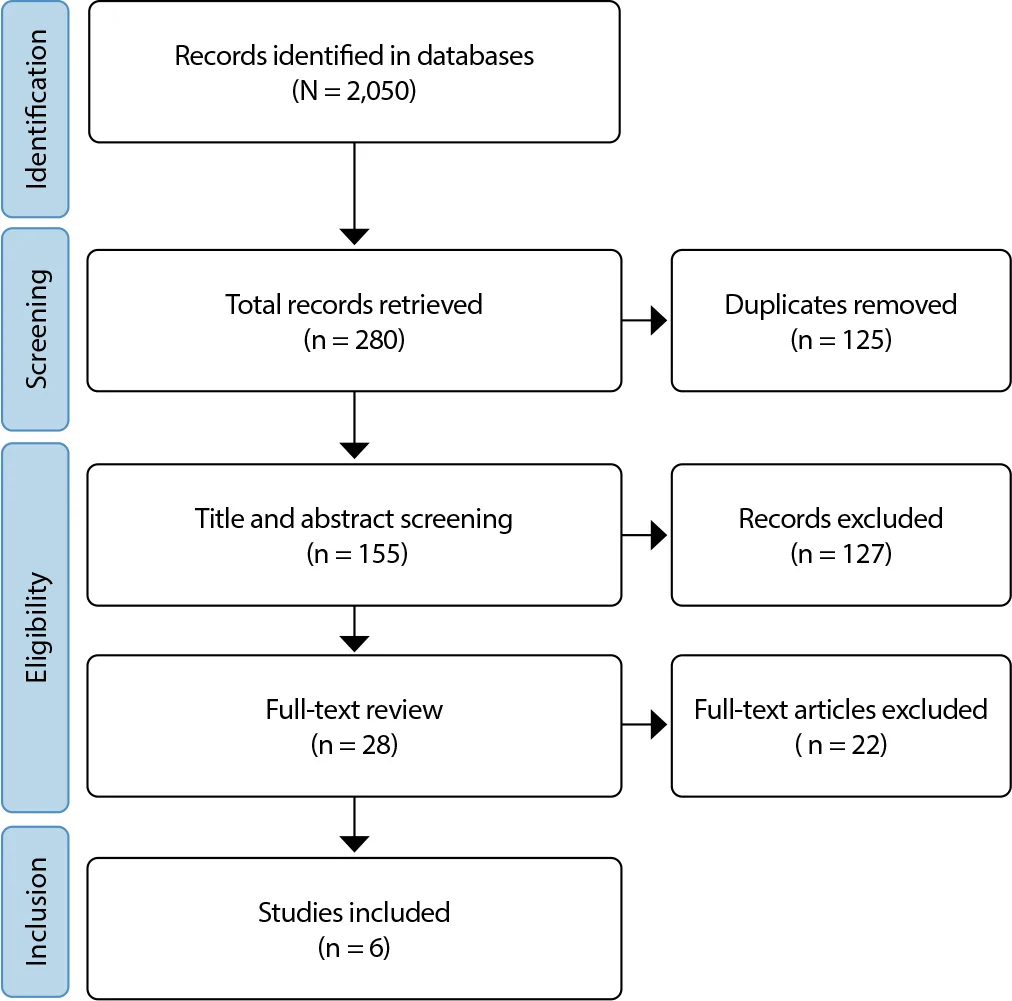
Flow diagram of the study selection process, São Paulo, São Paulo, Brazil, 2025

We identified 280 English-language records across eight databases. After removing 125 duplicates, 155 records underwent title and abstract screening; 127 were excluded. We assessed 28 full-text articles for eligibility and included 6 studies in the review (Figure 1).

Interrater agreement during screening was almost perfect (k = 0.93; p = 0.001) (Table 1).

**Table 1:** Interrater agreement (Cohen’s kappa), São Paulo, São Paulo, Brazil, 2025

Method	Cohen’s kappa coefficient for 2 raters (unweighted)
Records rated	155
Raters	2
Agreement %	99
Kappa	0.9303
z	11.58
*p* value	< 0.001

### Characteristics of included studies

To characterize the six included studies, we extracted data on authors, study title, year of publication, country, study design, number of participants, sex, intervention, and outcomes (Chart 1). The included studies were published between 2016 and 2023 and were conducted in Brazil^(29)^, Canada^(30)^, the Netherlands^(31)^, the United States^(32,33)^, and Iran^(34)^.

**Chart 1:** Summary of characteristics of included studies, São Paulo, São Paulo, Brazil, 2025

Author/Title	Year/ Country	Design/number of participants	Intervention	Outcome	Category
Danucalov et al.^(29)^ Yoga and compassion meditation program improve quality of life and self-compassion in family caregivers of Alzheimer’s disease patients: A randomized controlled trial	2017 Brazil	Randomized controlled trial 46 participants: 25 in the intervention group (3 men and 22 women) and 21 in the control group (2 men and 19 women)	Yoga and compassion meditation program 8 weeks; three 1 h 15 min sessions per week	Quality of life, mindfulness, vitality, and self-compassion	Not applicable
Goodridge et al.^(30)^ An app-based mindfulness-based self-compassion program to support caregivers of people with dementia: Participatory feasibility study	2021 Canada	Two-phase participatory feasibility study with a pretest-posttest design 29 participants: 3 men and 26 women	Mobile app delivering a mindfulness-based self-compassion intervention during 12 weeks	Changes in coping styles and emotional well-being	Acceptability, practicality, and implementation (feasibility study)
Berk et al.^(31)^ Mindfulness-Based Intervention for People With Dementia and Their Partners: Results of a Mixed-Methods Study.	2019 Netherlands	Mixed-methods pilot study 14 participants: 7 men (2 people with dementia and 5 caregivers) and 7 women (5 people with dementia and 2 caregivers)	Attention training program for people with dementia and their caregivers 9 weeks; eight weekly 2 h 30 min sessions and one day of silence	Improved quality of life, mindfulness, and positive mental health Reduced psychological distress (stress, anxiety, and depressive symptoms), worry, and perceived burden	Experiences; participation in the group with people with dementia and their caregivers; perceived effects; and practical feasibility
Yang et al.^(32)^ How family dementia caregivers perceive benefits of a 4-week Mentalizing Imagery Therapy program: a pilot study.	2022 United States	Qualitative feasibility pilot study 11 participants (2 men and 9 women)	Mentalizing Imagery Therapy 4 weeks; weekly 2-hour sessions	Not applicable	Perceived improvements in psychological well-being, interpersonal relationships, and self-compassion
Han et al.^(33)^ Effects of a compassion-based program on the grief experienced by caregivers of people suffering from dementia: a randomized controlled clinical trial.	2023 United States	Pilot randomized controlled trial 19 participants (all women)	Acceptance and Commitment Therapy delivered via videoconference 8 weeks; weekly 1-hour sessions	Reduced psychological distress (stress, anxiety, and depressive symptoms), perceived burden, grief, guilt, and cognitive fusion Improved quality of life, psychological flexibility, the meaningfulness of activities of daily living, and self-compassion	Not applicable
Jahani et al.^(34)^ Effects of a compassion-based program on the grief experienced by caregivers of people suffering from dementia: a randomized controlled clinical trial.	2022 Iran	Randomized controlled trial 70 participants: 35 in the intervention group (8 men and 27 women) and 35 in the control group (4 men and 31 women)	Online compassion-based program Five weekly sessions	Grief experience; domains: personal sacrifice burden, profound sadness and longing, worry, and isolation	Not applicable

Regarding study design, four used quantitative designs^(29,32,33,34)^, one used a qualitative design^(32)^, and one used a mixed-methods design^(31)^. Most participants were family caregivers of people with dementia, except for one study that included both family caregivers and people with dementia^(31)^. Participants ranged from 18 to 81 years, and most were women.

### Interventions implemented

The included studies tested a range of interventions for family caregivers of people with dementia. Formats and content varied considerably, spanning online and in-person approaches and emphasizing mindfulness, compassion, yoga, mentalization, and Acceptance and Commitment Therapy (ACT). All included articles aimed to develop and/or implement interventions designed to promote positive health outcomes among family caregivers.

Five articles incorporated mindfulness or other meditative practices. Danucalov et al.^(29)^ proposed a low-intensity yoga program combined with compassion meditation and mindfulness practices, delivered over 8 weeks with three 1 h 15 min sessions per week. Yang et al.^(32)^ used Mentalizing Imagery Therapy, which focuses on balanced attention to mentalization through a series of mindfulness and guided imagery practices. The intervention consisted of four weekly 2-hour sessions and included stretching and breathing exercises. Goodridge^(30)^ developed a program grounded in mindfulness to support an app-based intervention. Over 12 weeks, participants received a mindfulness-based self-compassion intervention supplemented with materials such as podcasts and guided meditations.

Han et al.^(33)^ delivered ACT, which uses mindfulness practices as a therapeutic tool. Sessions were held via videoconference over eight weekly 1-hour sessions, with individualized support and online resources for participants.

Berk et al.^(31)^ implemented the TANDEM program (named after a Dutch-language acronym referring to Attention Training for People with Dementia and Their Caregivers), inspired by Mindfulness-Based Stress Reduction (MBSR). The program comprised eight weekly 2 h 30 min sessions, combining meditation practices with facilitated discussions focusing on acceptance and communication. Similarly, Jahani et al.^(34)^ delivered an online compassion-based program comprising five weekly sessions that included breathing exercises and acceptance techniques.

### Study findings

Across the included studies, which tested different intervention approaches (mindfulness, yoga, ACT, and mentalization), interventions were associated with favorable psychosocial outcomes among family caregivers, including improved emotional well-being and quality of life, decreased anxiety, and increased acceptance. Improvements were also noted in grief-related outcomes and interpersonal relationships.

Among the three articles reporting improvements in self-compassion, one was qualitative, and two were randomized controlled trials. Danucalov et al.^(29)^ found improvements in self compassion, quality of life, attention, and vitality. After the yoga intervention, the intervention group scored higher than the control group on the self-compassion scale, particularly on the self kindness subscale. In the ACT study, the intervention group had higher self-compassion scores at 1-month follow-up than at baseline^(33)^. The study also reported significant decreases in grief and anxiety, along with increases in quality of life and psychological flexibility. In Yang et al.^(32)^, participants reported improvements in self-compassion, forgiveness, and their ability to manage challenging emotions after the intervention; they also reported enhanced decision-making ability and a greater ability to find comfort within themselves.

Although some studies did not show increases in self-compassion, other beneficial effects were observed. The TANDEM program was associated with increased calm and relaxation, greater acceptance of the dementia diagnosis, and improved self-care. Participants reported a stronger sense of connection with the group and improved relationships, along with decreased loneliness and interpersonal conflict. Quantitatively, Berk et al.^(31)^ reported increased quality of life and mindfulness. Jahani et al.^(34)^ found significant decreases in grief scores, profound sadness and longing, worries, and isolation, suggesting that the intervention helped alleviate grief related feelings.

Goodridge et al.^(30)^ did not find increased self-compassion; however, analyses indicated increased emotional well-being and decreased emotion-focused coping. Caregiver burden was negatively correlated with emotional well-being and positively correlated with avoidance-based coping and with the number of behaviors reported for the person with dementia. Participants predominantly used emotion-focused and acceptance-based coping strategies.

## DISCUSSION

The six studies included in this scoping review reported improvements in emotional well being and quality of life, and three reported increases in self-compassion. The interventions examined included yoga, mindfulness, ACT, self-compassion-based therapy, and mentalization. Two of the studies showing improvements in self-compassion were randomized controlled trials: one evaluated ACT, and the other evaluated yoga combined with compassion meditation. Although the evidence base is very small, these findings suggest that some interventions may help foster self compassion, well-being, and quality of life.

This scoping review, to our knowledge, is the first to examine this topic and may help inform care policies for family caregivers of people with dementia. However, several limitations should be considered. In addition to the limited number of studies, comparisons were constrained by substantial methodological heterogeneity. Only two studies used randomized controlled designs, and their interventions differed markedly. Overall, the interventions were heterogeneous, though they shared elements predominantly focused on contemplation. As a result, we could not determine which specific components may be most promising for promoting the development of self-compassion, limiting conclusions to broader inferences.

All interventions that showed improvements in self-compassion incorporated mindfulness practices. Serrão et al.^(35)^ suggest that increased mindfulness is associated with higher levels of self-compassion. In the study by Paucsik et al.^(36)^, mindfulness was also reported to increase self-compassion. In addition, higher levels of mindfulness and self-compassion are associated with lower psychological distress^(37,38)^. As argued by Neff^(39)^, mindfulness is a prerequisite for self compassion.

Mindfulness is one of the pillars of self-compassion because it enables people to stay present in the here and now with openness, cultivating the ability to notice sensations, emotions, and thoughts without resistance or avoidance^(40)^. According to Neff^(40)^, people need to recognize suffering and be aware of it to respond with kindness. In addition, as individuals become more attuned to their feelings and needs, they may also develop greater compassion for their own and others’ suffering^(41)^.

The literature suggests that yoga interventions have improved self-compassion subscales in other populations. Kinchen et al.^(42)^ observed improvements in the self-kindness subscale among nurses after a yoga intervention, whereas Miyoshi et al.^(43)^ reported improvements in the common humanity subscale among physicians. Snaith^(44)^ found a positive correlation between yoga and self-compassion among long-term yoga practitioners^(45)^. In addition, Pattel^(46)^ identified improvements in self-compassion scores after yoga was implemented among university students.

Consistent with Yang et al.^(32)^, the randomized controlled trial conducted by Jain^(47)^ showed increased self-compassion after 4 weeks of mentalization-based therapy. Although few studies have examined ACT for developing self-compassion, some research has reported significant improvements after the intervention. The cognitive flexibility developed through ACT is an important mediator of self-compassion development because it encompasses skills such as acceptance of emotions and thoughts and mindfulness^(48)^, aligning with Han et al.^(33)^.

### Study limitations

As a scoping review, this study may have excluded relevant publications due to the inclusion criteria adopted. In addition, the diversity of methods and interventions across the selected studies may hinder the synthesis and interpretation of intervention benefits for self-compassion development.

Future studies are needed to deepen knowledge about the elements and specific features required to strengthen self-compassion. In addition, exploring differences between online and in person interventions may offer valuable insights for applying these approaches to support caregivers and people with dementia. Research is also limited regarding the benefits of self compassion in promoting well-being.

### Contributions to the field of nursing

Given the importance of interventions that support mental and emotional health, nursing plays a key role in care delivery, in supporting patients, and in providing user embracement and guidance to caregivers. These professionals are essential for identifying caregivers’ needs and implementing support strategies^(49,50)^. In this setting, caregivers’ psychological distress is a recurring concern in routine care.

The findings of this review have relevant implications for nursing practice, particularly in health care for family caregivers and in mental health care. Therefore, this study reinforces the importance of broadening nursing’s focus to include care strategies that support family caregivers of people with dementia in their mental and emotional health.

## CONCLUSIONS

Although few studies were identified in this review, the available literature indicates that some interventions may help foster self-compassion and increase well-being among caregivers of people with dementia. ACT and yoga combined with compassion meditation stood out as the most promising interventions for promoting self-compassion in the studied population.

## Data Availability

The research data are available only upon request.
