# Supplementary material for: Self-compassion interventions for family caregivers of people with dementia: a scoping review
Source: Rev Bras Enferm. 2026 Aug 21;79:e20250252. doi: 10.1590/0034-7167-2025-0252 (PMC13505566; doi:10.1590/0034-7167-2025-0252)
Supplement: Translation

# Translation: Autocompaixão em intervenções para familiares cuidadores de pessoas com demência: revisão de escopo

**DOI:** 10.1590/0034-7167-2025-0252pt

**Authors:** Marina Noto Faria, Joyce Salgado Santos, Fernanda Guilhermino Magalhães, Ana Karla Silva Soares, Ana Lucia de Moraes Horta

**Affiliations:** I Universidade Federal de São Paulo. São Paulo, São Paulo, Brasil; II Universidade Federal do Mato Grosso do Sul. Campo Grande, Mato Grosso do Sul, Brasil

**Keywords:** Demência, Cuidador, Compaixão, Intervenções Psicossociais, Revisão de Escopo.

## Abstract

**Objetivos:**

identificar intervenções com potencial de desenvolver autocompaixão entre cuidadores familiares de pessoas com demência.

**Métodos:**

realizou-se uma revisão de escopo em oito bases de dados, com os descritores *dementia, self-compassion, empathy, family, caregivers* e *mental health*. Incluíram-se artigos em inglês, abordando intervenções baseadas em atenção plena e/ou compaixão. Três revisores fizeram a seleção com auxílio do software Rayyan.

**Resultados:**

dos 280 artigos encontrados, 6 foram incluídos. As intervenções envolveram ioga, *mindfulness*, Terapia de Aceitação e Compromisso, terapia baseada em autocompaixão e mentalização. Três estudos relataram aumento da autocompaixão.

**Conclusões:**

a literatura aponta que algumas intervenções podem favorecer o desenvolvimento da autocompaixão entre cuidadores de pessoas com demência.

## INTRODUÇÃO

O *Diagnostic and Statistical Manual of Mental Disorders* (DSM-V) classifica a demência como uma doença neurodegenerativa que consiste no declínio cognitivo progressivo, ocasionando diminuição significativa da capacidade intelectual e cognitiva^(1)^ O declínio das funções cognitivas pode comprometer habilidades necessárias para realizar as atividades cotidianas. Com o agravamento da doença, as pessoas com demência passam a depender cada vez mais dos cuidados de terceiros^(2)^_._

Muitas vezes, os familiares são os cuidadores principais das pessoas com demência^(2)^. Sendo assim, o aumento progressivo da oferta de cuidado pode gerar consequências importantes na saúde desses cuidadores. Tal população pode apresentar sobrecarga emocional, fadiga, alterações do sono, ansiedade, depressão, níveis elevados de estresse, dores nas costas, *caregiver burden* e estigmatização^(2-6)^.

Diante desse contexto, diversos estudos focados nas necessidades desses familiares foram desenvolvidos. Uma das principais demandas identificadas foi a partilha de suas experiências, preocupações e satisfações referentes ao processo de cuidado, além da necessidade de desenvolver estratégias de coping para melhorar a saúde física e mental^(2,7-9)^.

A autocompaixão é uma habilidade relacionada ao bem-estar e à redução de pensamentos negativos autorreferidos, ansiedade, depressão, estresse, ruminação, evitação, supressão de pensamentos e vergonha, bem como à diminuição do impacto psicológico do estigma internalizado^(10-17)^. Conceitualmente, configura-se como a sensibilidade ao próprio sofrimento, acompanhada do compromisso de tentar aliviá-lo.^(18,19)^ É composta por três pilares: autobondade, *mindfulness* e humanidade compartilhada^(18)^. A autobondade refere-se à capacidade de ser “amoroso” consigo mesmo em meio ao sofrimento; o *mindfulness* significa estar consciente de pensamentos e sentimentos desafiadores, em vez de se identificar demais com eles^(20)^; e a humanidade compartilhada é o reconhecimento da interconectividade entre os seres^(19)^.

Em razão da ampliação do conhecimento sobre autocompaixão e seus benefícios para a saúde mental, diversos estudos na literatura nacional e internacional foram realizados aplicando o *mindfulness* e outras práticas envolvendo autocompaixão em familiares de pessoas com demência; porém, são poucas as revisões que abordam o impacto de tais intervenções na saúde mental dessa população^(14,15)^.

## OBJETIVOS

Identificar intervenções com potencial de desenvolver autocompaixão entre cuidadores familiares de pessoas com demência.

## MÉTODOS

Esta revisão de escopo foi conduzida conforme a metodologia do JBI e relatada de acordo com o checklist PRISMA-ScR^(21,22)^. A pergunta norteadora definida para a revisão foi: “Quais são os tipos de intervenções disponíveis com potencial de desenvolver autocompaixão em cuidadores familiares de pessoas com demência e quais são seus impactos na saúde mental?”. O protocolo foi registrado no Open Science Framework (OSF), disponível em OSF.IO/9P8RV (doi: 10.17605).

A presente revisão de escopo seguiu estas etapas: definição da pergunta de pesquisa; definição das palavras-chave e estratégia de busca; seleção dos estudos; e leitura dos artigos para avaliação. Foram utilizadas oito bases de dados: PubMed (*National Center For Biotechnology Information*), Biblioteca Virtual de Saúde (BVS), EMBASE, ProQuest, PsycINFO, Latin American and Caribbean Health Sciences Literature (LILACS), Education Resources Information Center (ERIC) e Cumulative Index to Nursing and Allied Health Literature (CINAHL). De acordo com cada base de dados, foram utilizados determinados filtros, tais como data da pesquisa, textos completos disponíveis, artigos originais e pesquisa com humanos. Foram incluídos estudos disponíveis em inglês. Artigos indisponíveis e de literatura cinzenta não foram incluídos na revisão. Nenhuma limitação de data foi aplicada. A pesquisa foi realizada entre dezembro de 2023 e setembro de 2024.

A busca incluiu descritores de acordo com a base de dados (DeCS e MeSH): *dementia, self-compassion, empathy, compassion, family, caregivers* e *mental health*. Esta foi a estratégia de busca para identificação de documentos: (“*Dementia*”) *AND* (“*caregivers*”) *AND* (“*Family*” *OR* “*partner*” *OR* “*family members*” *OR* “*members*” *OR* “*relatives*”) *AND* (“*self compassion*” *OR* “*empathy*” *OR* “*compassion*” *OR* “*mindfulness*” *OR* “*mindfulness based stress reduction*” *OR* “*mindfulness based stress reduction*” *OR* “*mindfulness*-*based cognitive therapy*” *OR* “*acceptance and commitment therapy*” *OR* “*meditation*” *OR* “loving kindness meditation*” *OR* “*compassion meditation*” *OR* “*mindful self compassion*” *OR* “*compassion focused therapy*” *OR* “*compassion cultivation training*” *OR* “*cultivating emotional balance*).

### Critérios de inclusão

(1) artigos originais, completos, publicados em inglês e em periódico com revisão por pares (2) estudos sobre intervenções envolvendo compaixão ou autocompaixão em familiares de pessoas com demência, (3) amostra composta somente por familiares cuidadores de pessoas com demência ou familiares cuidadores e a(s) pessoa(s) com demência, (4) estudos que avaliassem as respostas psicológicas (níveis de compaixão, autocompaixão, ansiedade, depressão, *caregiver burden*, qualidade de vida, bem-estar, entre outros) nos familiares de pessoas com demência e (5) pesquisas com desenhos de estudos controlados randomizados e não randomizados, estudos experimentais e quase experimentais, bem como estudos qualitativos. O artigo devia envolver familiares de pessoas com demência e apresentar intervenção baseada em atenção plena e/ou compaixão.

### Critérios de exclusão

(1) pesquisas publicadas em formato de carta, dissertação de mestrado, artigos teóricos e resumos estendidos e/ou tese de doutorado. (2) estudos que não apresentaram avaliação antes e depois da intervenção (exceto em casos de estudos qualitativos).

### Coleta, organização e análise dos dados

Dois revisores independentes fizeram a seleção e a triagem dos estudos. As referências foram gerenciadas no *software* Rayyan^(23)^, e os duplicados foram removidos. Em seguida, títulos e resumos foram avaliados; os registros que não atenderam aos critérios de elegibilidade foram excluídos, com o motivo da exclusão devidamente registrado. Posteriormente, os revisores avaliaram, de forma independente, os textos completos. Ao longo da triagem, foram realizadas reuniões quinzenais para alinhamento. Divergências foram resolvidas por consenso e, quando necessário, um terceiro revisor foi acionado.

A avaliação da concordância entre os avaliadores se deu por meio do índice de concordância Kappa, calculado no software Jamovi^(24)^. Para interpretação do Kappa, foram utilizadas as seguintes categorias: Leve (até 0,20); Pequena (0,21 a 0,40); Moderada (0,41 a 0,60); Substancial (0,61 a 0,80); Quase perfeita (superior a 0,81)^(25,26)^.

Para avaliar o risco de viés, foi adotado o checklist *JBI Critical Appraisal Tools.* Para aqueles que apresentavam métodos que não tinham avaliação do JBI, foram usados os checklists da EQUATOR Network. O *JBI Critical Appraisal Tools* envolve uma série de perguntas sobre a metodologia utilizada no artigo. Cada item apresenta as seguintes opções de resposta: “Sim”, “Não”, “Não fica claro” ou “Não aplicável”^(21)^. Com base nas respostas, é possível avaliar a qualidade metodológica. Quanto maior a quantidade de “Sim”, melhor a qualidade do estudo; quanto maior a quantidade de “Não” ou “Não ficou claro”, menor é sua conformidade com as normas metodológicas solicitadas^(27)^. Dos checklists da EQUATOR Network, utilizou-se o STROBE (*Strengthening the reporting of observational studies in epidemiology*) para avaliar um dos artigos^(28)^. Trata-se de uma diretriz de relato composta por 22 itens que devem ser contemplados na redação do manuscrito, com o objetivo de padronizar e aprimorar a transparência do relato. Quanto maior o número de itens atendidos, mais completo o relato do estudo. No entanto, o artigo avaliado com o STROBE foi excluído do estudo por não atender aos requisitos metodológicos estabelecidos^(22)^.

## RESULTADOS

Na presente pesquisa, 280 estudos escritos em inglês foram identificados em oito bases de dados. Posteriormente, foram removidos 125 estudos duplicados. Um total de 155 passou pela triagem de título e resumo, e 127 foram excluídos. Foi revisado o texto completo de 28 estudos. No fim da triagem, seis artigos foram incluídos na revisão (Figura 1).

**Figura 1:** Fluxograma com as etapas de seleção dos artigos, São Paulo, São Paulo, Brasil, 2025

O coeficiente de Kappa utilizado para avaliar a concordância dos revisores no processo de triagem apresentou uma concordância considerada “quase perfeita” (k = 0,93; p = 0,001) (Tabela 1).

**Tabela 1:** Coeficiente de concordância Kappa, São Paulo, São Paulo, Brasil, 2025

Método	Coeficiente Kappa de Cohen para 2 avaliadores (Peso: não ponderado)
Itens avaliados	155
Avaliadores	2
Concordância %	99
Kappa	0,9303
z	11,58
Valor de *e*	< 0,001

### Características do estudo

As variáveis utilizadas para caracterizar os seis artigos incluídos foram: autores, título do estudo, ano de publicação, país, desenho do estudo, número de pacientes, gênero, intervenção e desfechos (Quadro 1). Após a análise, foram identificados estudos publicados entre 2016 e 2023 e realizados no Brasil^(29)^, Canadá^(30)^, Holanda^(31)^ Estados Unidos^(32,33)^ e Irã^(34)^

**Quadro 1:** Resumo das características dos estudos incluídos, São Paulo, São Paulo, Brasil, 2025

Autor/Título	Ano/ País	Desenho/número de pacientes	Intervenção	Desfecho	Categoria
Danucalov et al.^(29)^ *Yoga and compassion meditation program improve quality of life and self-compassion in family caregivers of Alzheimer’s disease patients: A randomized controlled trial*	2017 Brasil	Ensaio clínico randomizado controlado 46 participantes - 25 no grupo experimental (3 homens e 22 mulheres) e 21 no grupo-controle (2 homens e 19 mulheres)	Programa de ioga e meditação compassiva; 8 semanas; 3 encontros semanais de 1h 15min.	Qualidade de vida, atenção plena, vitalidade e autocompaixão	Não se aplica
Goodridge et al.^(30)^ *An app-based mindfulness-based self-compassion program to support caregivers of people with dementia: Participatory feasibility study*	2021 Canadá	Estudo de viabilidade participativo pré-teste e pós-teste, em duas fases 29 participantes - 3 homens e 26 mulheres	Aplicativo móvel com intervenção de autocompaixão baseada na atenção plena - duração de 12 semanas	Mudanças nos estilos de enfrentamento e bem-estar emocional	Aceitabilidade, praticidade e implementação (estudo de viabilidade)
Berk et al.^(31)^ *Mindfulness-Based Intervention for People With Dementia and Their Partners: Results of a Mixed-Methods Study.*	2019 Holanda	Estudo-piloto de métodos mistos 14 participantes - 7 homens (2 pessoas com demência e 5 cuidadores) e 7 mulheres (5 pessoas com demência e 2 cuidadores)	Programa de treinamento de atenção para pessoas com demência e seus cuidadores 9 semanas; 8 sessões semanais de 2h 30min e um dia de silêncio	Melhora da qualidade de vida, atenção plena e saúde mental positiva Diminuição do sofrimento psicológico (estresse, ansiedade e sintomas depressivos), preocupação e sobrecarga percebida	Experiências, participação no grupo com pessoas com demência e seus cuidadores, efeitos e viabilidade prática
Yang et al.^(32)^ *How family dementia caregivers perceive benefits of a 4-week Mentalizing Imagery Therapy program: a pilot study.*	2022 Estados Unidos	Estudo-piloto de viabilidade (qualitativo) 11 participantes (2 homens e 9 mulheres)	Terapia de mentalização de imagens 4 semanas; sessões semanais 2 horas	Não se aplica	Percepção de melhora do bem-estar psicológico, dos relacionamentos interpessoais e da autocompaixão
Han et al.^(33)^ *Effects of a compassion-based program on the grief experienced by caregivers of people suffering from dementia: a randomized controlled clinical trial.*	2023 Estados Unidos	Estudo-piloto de um ensaio clínico randomizado controlado 19 participantes do sexo feminino	Terapia de Aceitação e Compromisso por videoconferência 8 semanas; sessões semanais de 1 hora	Diminuição do sofrimento psicológico (estresse, ansiedade e sintomas depressivos), sobrecarga percebida, luto, culpa e fusão cognitiva Melhora da qualidade de vida, flexibilidade psicológica, significância das atividades cotidianas e autocompaixão	Não se aplica
Jahani et al.^(34)^ *Effects of a compassion-based program on the grief experienced by caregivers of people suffering from dementia: a randomized controlled clinical trial.*	2022 Irã	Ensaio clínico randomizado controlado 70 participantes - 35 no grupo experimental (8 homens e 27 mulheres) e 35 no grupo-controle (4 homens e 31 mulheres)	Programa on-line baseado na compaixão 5 sessões semanais	Experiência de luto; campos de carga de sacrifício pessoal, tristeza e saudade profundas, preocupação e isolamento	Não se aplica

Quanto ao desenho, quatro adotaram métodos quantitativos^(29,32-34)^, um utilizou método qualitativo^(32)^ e um empregou método misto^(31)^. A maioria dos participantes eram familiares de pessoas com demência, com exceção de um estudo que incluiu os familiares cuidadores e as pessoas com demência^(31)^. Os participantes dos estudos tinham entre 18 a 81 anos, com predominância do sexo feminino.

### Intervenção aplicada

Os estudos aplicaram diferentes intervenções com familiares de pessoas com demência. Houve variação de formato e conteúdo, abrangendo métodos on-line e presenciais, com enfoques em *mindfulness*, compaixão, ioga, mentalização e Terapia de Aceitação e Compromisso (ACT). Todos os artigos incluídos tiveram como objetivo a criação e/ou aplicação de intervenções voltadas a promover desfechos positivos de saúde entre os familiares.

Cinco artigos aplicaram intervenções envolvendo *mindfulness* ou práticas meditativas em sua estrutura. O estudo de Danucalov et al.^(29)^ propôs um programa de ioga de baixa intensidade associado à meditação compassiva e práticas de *mindfulness*, com duração de oito semanas e três encontros semanais de 1h 15min. Yang et al.^(32)^ utilizaram a terapia de mentalização de imagens, que consiste na atenção equilibrada à mentalização em uma série de práticas de atenção plena e de imagens guiadas. Foram quatro sessões semanais de duas horas, envolvendo exercícios de alongamento e respiração. O estudo realizado por Goodridge^(30)^ também adotou programas ancorados em *mindfulness* para desenvolver um aplicativo. Durante 12 semanas, foi realizada uma intervenção com base em autocompaixão, incorporando materiais de suporte como podcasts e meditações.

O estudo de Han et al.^(33)^ aplicou a ACT, que usa práticas de atenção plena como ferramenta terapêutica. Os encontros ocorreram por videoconferência, com duração de oito sessões semanais de uma hora, suporte individualizado e recursos on-line para os participantes.

Já o estudo de Berk et al.^(31)^ implementou o programa TANDEM (assim denominado por ser um acrônimo em holandês que se refere ao Treinamento de Atenção para Pessoas com Demência e seus Cuidadores), inspirado no *Mindfulness-Based Stress Reduction* (MBSR). Foram oito sessões semanais de 2h 30min, abordando práticas de meditação e discussão sobre aceitação e comunicação. Na mesma linha, o estudo Jahani et al.^(34)^ desenvolveu discussões e práticas de aceitação. Foi aplicado um programa on line de cinco sessões semanais, focalizando a compaixão e incluindo exercícios de respiração e técnicas de aceitação.

## RESULTADOS

Os estudos incluídos, que empregaram diferentes abordagens de intervenção (*mindfulness*, ioga, ACT e mentalização), relataram efeitos favoráveis em desfechos psicossociais dos familiares, com melhorias no bem-estar emocional e na qualidade de vida, além de redução de ansiedade e aumento de aceitação. Também foram observadas melhorias no que se refere ao luto e aos relacionamentos interpessoais.

Entre os três artigos que apresentaram melhoras na autocompaixão, um era qualitativo e dois eram ensaios clínicos randomizados controlados. Danucalov et al.^(29)^ observaram melhoras nos níveis de autocompaixão, qualidade de vida, atenção e vitalidade. Após a intervenção de ioga, o grupo-intervenção obteve melhores escores do que o grupo-controle na escala de autocompaixão, principalmente na subescala de autobondade. Em estudo que aplicou a ACT, o grupo intervenção teve melhores escores de autocompaixão após um mês de acompanhamento em relação ao pré teste^(33)^. Observou-se também redução significativa no luto e ansiedade, bem como aumento da qualidade de vida e flexibilidade psicológica. No estudo de Yang et al.^(32)^, os participantes relataram melhorias na autocompaixão, perdão e manejo de emoções desafiadoras após a intervenção. Eles também destacaram a capacidade aprimorada de tomar decisões e encontrar conforto em si mesmos.

Embora alguns estudos não tenham evidenciado elevação nos níveis de autocompaixão, foram observados outros efeitos benéficos após as intervenções. O programa TANDEM proporcionou benefícios como aumento da calma e relaxamento, aceitação do diagnóstico de demência e melhora no autocuidado. Os participantes relataram uma maior sensação de conexão com o grupo e melhorias nos relacionamentos, bem como uma redução na solidão e conflitos interpessoais. Quanto aos resultados quantitativos, houve um aumento nos níveis de qualidade de vida e na atenção plena Berk et al.^(31)^. O estudo desenvolvido por Jahani et al.^(34)^ resultou em uma redução significativa nos escores de luto, tristeza e saudade profundas, diminuição das preocupações e isolamento, evidenciando a eficácia da intervenção em aliviar sentimentos de luto.

O estudo realizado por Goodridge et al.^(30)^ não observou elevação nos níveis de autocompaixão, porém a análise revelou aumento do bem-estar emocional e diminuição do enfrentamento baseado na emoção. A sobrecarga dos cuidadores apresentou correlação negativa com o bem-estar emocional, ao passo que mostrou correlação positiva com enfrentamento baseado na evitação e com o número de comportamentos apresentados pelas pessoas com demência. Os participantes utilizaram predominantemente estratégias de enfrentamento com foco na emoção e aceitação.

## DISCUSSÃO

Os seis estudos identificados nesta revisão apontaram melhora do bem-estar emocional e qualidade de vida. Em três deles, houve aumento dos níveis de autocompaixão. As intervenções estudadas incluíram ioga, *mindfulness*, ACT, terapia baseada em autocompaixão e mentalização. Dois dos estudos que evidenciaram melhora na autocompaixão eram randomizados controlados, um dos quais utilizava ACT, e o outro adotava ioga com meditação compassiva. Embora em número muito limitado, tais achados indicam que algumas intervenções podem favorecer o desenvolvimento de autocompaixão, bem-estar e qualidade de vida.

A presente revisão de escopo é pioneira na investigação do tema e pode ser útil para subsidiar as políticas de cuidados dirigidos a familiares cuidadores de pessoas com demência. Porém, algumas limitações merecem consideração. Além da escassez de estudos, foi observada uma dificuldade de comparação por causa da grande diversidade metodológica. Apenas dois estudos adotaram desenho randomizado controlado, mas com intervenções substancialmente distintas. De modo geral, as intervenções foram heterogêneas, embora compartilhassem alguns elementos, predominantemente de natureza contemplativa. Assim, não foi possível diferenciar em profundidade quais componentes específicos seriam mais promissores para promover o desenvolvimento da autocompaixão, permitindo apenas inferências mais genéricas.

Todas as intervenções que demonstraram melhorias nos níveis de autocompaixão incluíram, em sua estrutura, práticas relacionadas ao *mindfulness*. Serrão et al.^(35)^ sugerem que o aumento de *mindfulness* está relacionado com a elevação dos níveis de autocompaixão. No estudo realizado por Paucsik et al.^(36)^, a atenção plena demonstrou aumentar a autocompaixão. Além disso, níveis mais altos de atenção plena e autocompaixão estão relacionados a menores níveis de sofrimento psicológico^(37,38)^. Conforme argumenta Neff^(39)^, o *mindfulness* é um pré-requisito da autocompaixão.

*Mindfulness* é um dos pilares da autocompaixão, porque permite estar presente no aqui e agora, com aberturas, desenvolvendo a habilidade de ter consciência das sensações, emoções e pensamentos sem resistência ou esquiva^(40)^. De acordo com Neff^(40)^, é preciso ser capaz de reconhecer o sofrimento e ter consciência dele para conseguir responder-lhe com bondade. Além disso, à medida que as pessoas entram em contato com os seus sentimentos e necessidades, vão também desenvolvendo a habilidade de compaixão com o seu próprio sofrimento e com o do outro^(41)^.

A literatura indica que intervenções de ioga demonstraram melhorias nas subescalas de autocompaixão em outras populações. O estudo desenvolvido por Kinchen et al.^(42)^ observou melhorias na subescala de autobondade entre profissionais de enfermagem pós-intervenção de ioga, enquanto Miyoshi et al.^(43)^ mostraram que médicos tiveram avanços na subescala de humanidade compartilhada. O estudo de Snaith^(44)^ encontrou uma correlação positiva entre ioga e autocompaixão em praticantes antigos de ioga^(45)^. Além disso, estudo realizado por Pattel^(46)^ identificou melhorias nos índices de autocompaixão após a aplicação de ioga em estudantes universitários.

Alinhado com os achados de Yang^(32)^, o ensaio clínico randomizado realizado por Jain^(47)^ demonstrou um aumento nos níveis de autocompaixão dos participantes após quatro semanas de terapia baseada em mentalização. Embora existam poucos estudos sobre a aplicação da ACT para o desenvolvimento da autocompaixão, algumas pesquisas indicam melhorias significativas após a intervenção. A flexibilidade cognitiva desenvolvida no processo da ACT é um mediador considerável para o desenvolvimento da autocompaixão, pois engloba diferentes habilidades, tais como aceitação das emoções e pensamentos e *mindfulness* ^(48)^ convergindo com o achado do estudo de Han et al.^(33)^.

### Limitações do estudo

Por ser uma revisão de escopo, destaca-se a possibilidade de exclusão de publicações relevantes devido aos critérios de inclusão adotados. Ademais, a multiplicidade de métodos e intervenções entre os trabalhos selecionados podem dificultar a síntese e a interpretação dos benefícios das intervenções no tocante ao desenvolvimento da autocompaixão.

São necessários futuros estudos para aprofundar o conhecimento sobre os elementos e/ou especificidades necessárias para fortalecer a autocompaixão. Além disso, explorar as diferenças entre intervenções on-line e presenciais pode oferecer insights valiosos para a aplicação dessas abordagens no suporte a cuidadores e pessoas com demência. Faltam investigações também sobre os benefícios da autocompaixão no processo de promoção do bem-estar.

### Contribuições para a área de Enfermagem

Considerando a importância de intervenções que promovam saúde mental e emocional, destaca-se o papel da enfermagem no cuidado, no suporte dos pacientes e no acolhimento e orientação dos cuidadores. Esses profissionais são fundamentais na identificação das necessidades dos cuidadores e na implementação de estratégias de suporte^(49,50)^. Nesse cenário, o sofrimento psicológico de cuidadores é uma preocupação recorrente nos atendimentos.

Os achados desta revisão apresentam implicações relevantes para a prática da enfermagem, sobretudo no contexto da atenção à saúde de cuidadores familiares e no cuidado em saúde mental. Portanto, este estudo contribui para reforçar a importância de se ampliar o olhar da enfermagem para estratégias de cuidado que promovam saúde mental e emocional para os cuidadores familiares de pessoas com demência.

## CONCLUSÕES

Apesar de terem sido identificados poucos estudos nesta revisão, a literatura disponível mostra que algumas intervenções têm potencial para favorecer o desenvolvimento da autocompaixão e aumentar o bem-estar entre cuidadores de pessoas com demência. ACT e ioga com meditação compassiva destacaram-se como as intervenções mais promissoras para promover autocompaixão na população estudada.

## Data Availability

Os dados de pesquisa só estão disponíveis mediante solicitação.
